# 4-[5-(4-Fluoro­phen­yl)-1,2-oxazol-4-yl]pyridine

**DOI:** 10.1107/S2414314625009873

**Published:** 2025-11-21

**Authors:** Pierre Koch, Dieter Schollmeyer, Stefan Laufer

**Affiliations:** a Universität Regensburg, Universitätsstrasse 31, 93053 Regensburg, Germany; b University Mainz, Department of Chemistry, Duesbergweg 10-14, 55099 Mainz, Germany; c Eberhard Karls Universität Tübingen, Department of Pharmaceutical/Medicinal Chemistry, Auf der Morgenstelle 8, 72076 Tübingen, Germany; Goethe-Universität Frankfurt, Germany

**Keywords:** crystal structure, pyridine, isooxazole, MAP kinase inhibitor

## Abstract

The title compound, C_14_H_9_FN_2_O, crystallizes in the monoclinic space group *P*2_1_/*c*. The dihedral angles between the central isoxazole ring and the 4-fluoro­phenyl and pyridine rings are 32.64 (5) and 32.70 (7)°, respectively.

## Graphical abstract

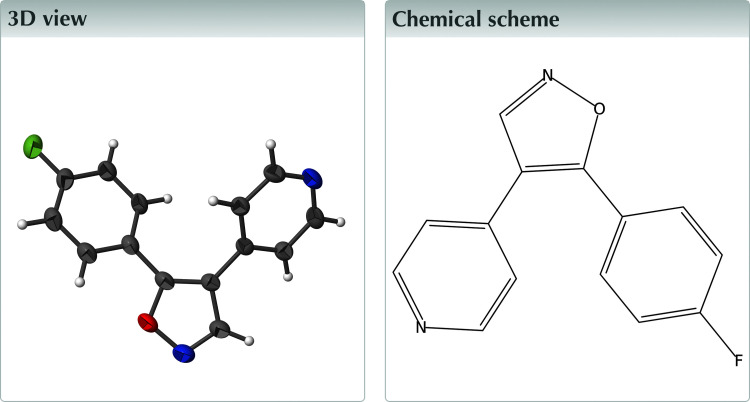

## Structure description

**Figure 1:**
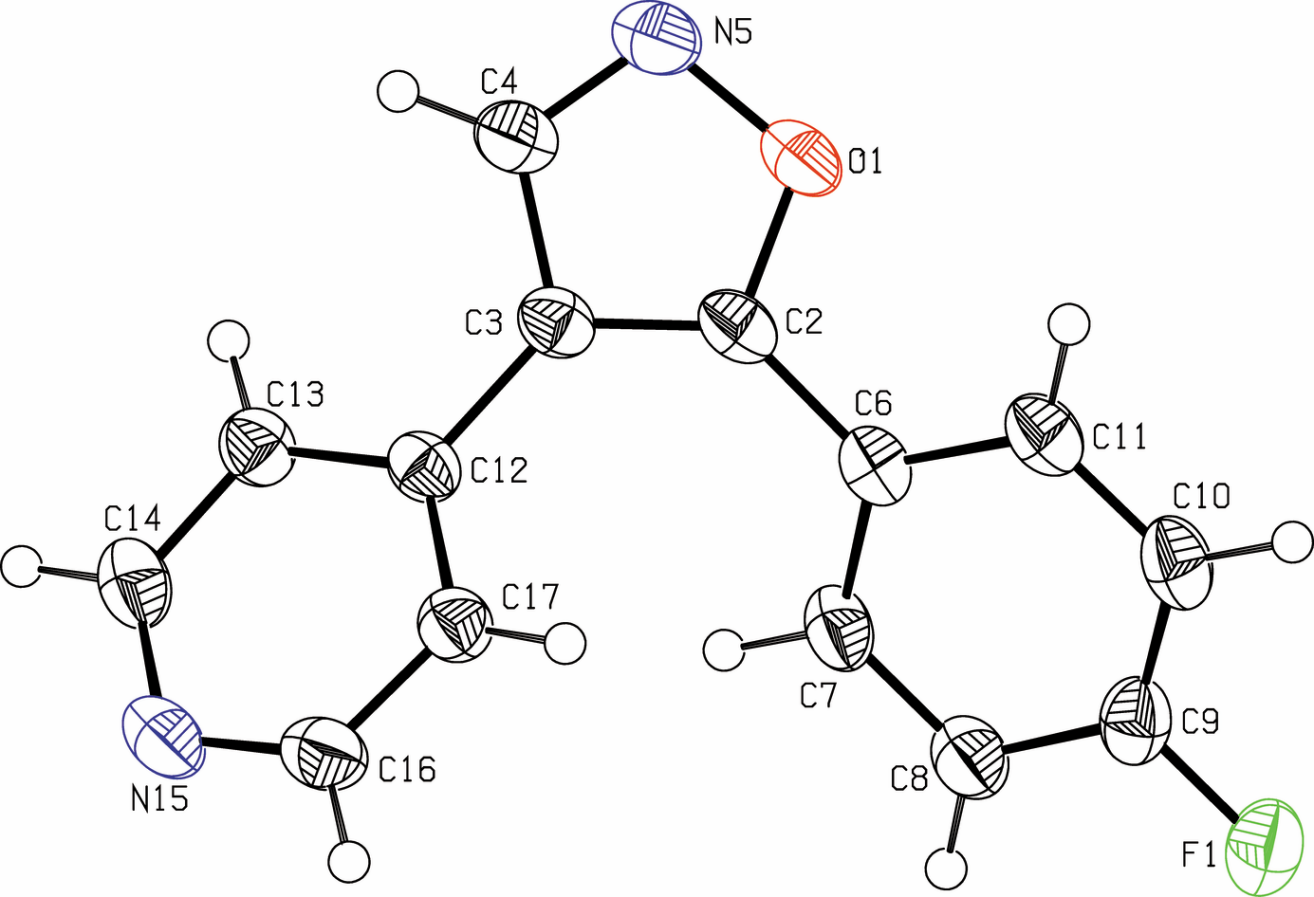
View of the title compound. Displacement ellipsoids are drawn at the 50% probability level.

**Figure 2:**
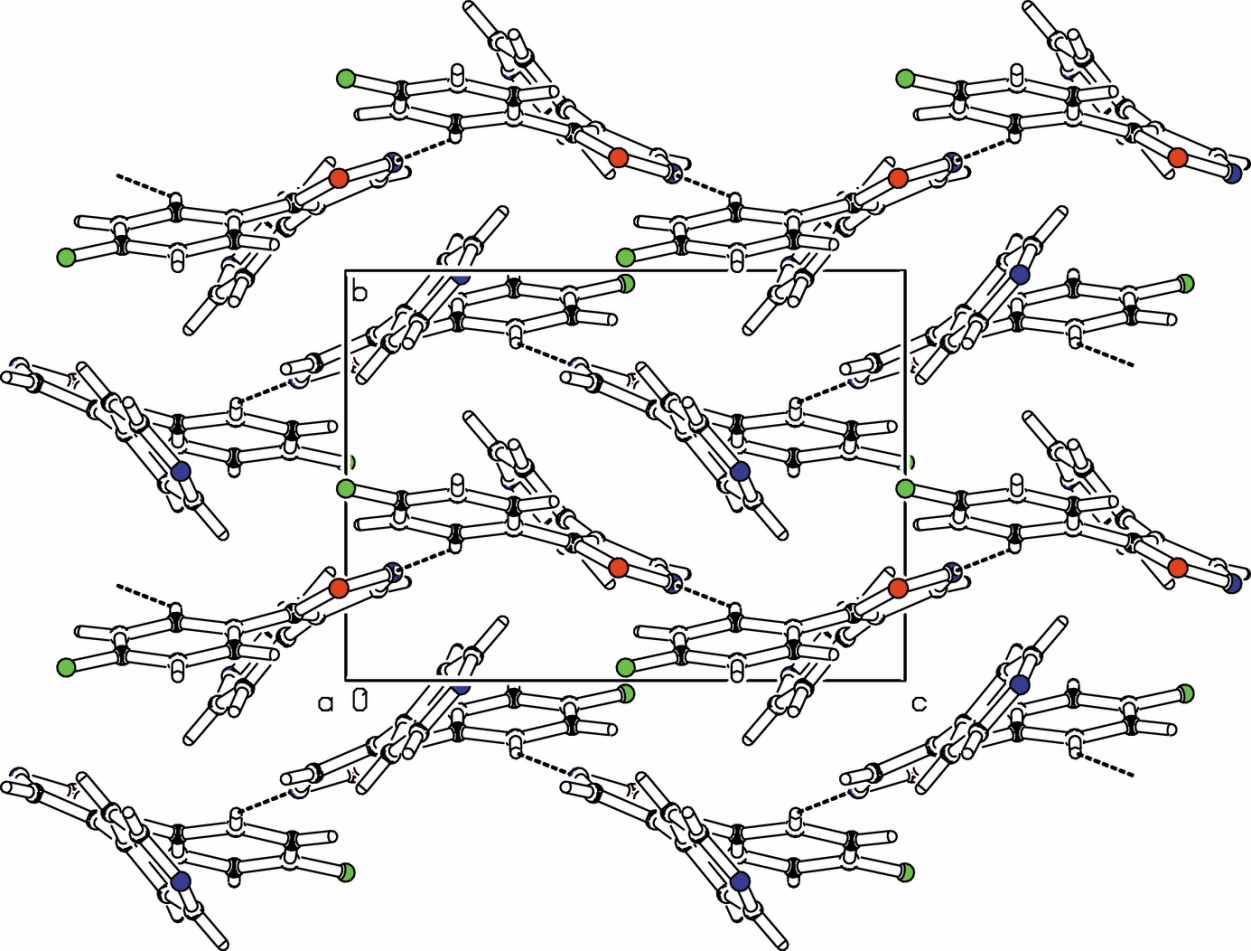
Part of the packing diagram. View along *a*-axis direction. Hydrogen bonds are drawn with dashed lines.

The title compound, C_14_H_9_FN_2_O (Fig. 1 ▸), was synthesized to extend our study on the role of the hydrogen-bonding heteroatom-Lys53 inter­action between pyridinyl-substituted five-membered heterocyclic ring inhibitors and the p38α mitogen-activated protein (MAP) kinase (Abu Thaher *et al.*, 2009 ▸). The isoxazole ring (O1,C2–C4,N5) makes dihedral angles of 32.64 (5) and 32.70 (7)° with the 4-fluoro­phenyl ring (C6–C11) and the pyridine ring (C12–C14, N15, C16, C17), respectively. The 4-fluoro­phenyl ring makes a dihedral angle of 46.37 (6)° with the pyridine ring. In the extended structure, C—H⋯N bonds link the mol­ecules into *C*(6) chains running along the *c*-axis direction (Table 1 ▸, Fig. 2 ▸).

## Synthesis and crystallization

The title compound was synthesized according to the protocol reported by Laufer *et al.* (2006 ▸). 500 mg of 3-(di­methyl­amino)-1-(4-fluoro­phen­yl)-2-(pyridin-4-yl)prop-2-en-1-one were dissolved in methanol (8 ml) and water (4 ml). 106 mg of sodium carbonate and 123 mg hydroxyl­amine hydro­chloride were added. The pH was adjusted to 5 by dropwise addition of acetic acid. The reaction mixture was heated to reflux temperature for 2.5 h. After cooling to room temperature, the pH was set to 7 by addition of ammonia. Ice was added to the mixture and the title compound slowly crystallized as a colorless solid (290 mg).

## Refinement

Crystal data, data collection and structure refinement details are summarized in Table 2 ▸.

## Supplementary Material

Crystal structure: contains datablock(s) I, global. DOI: 10.1107/S2414314625009873/bt4187sup1.cif

Structure factors: contains datablock(s) I. DOI: 10.1107/S2414314625009873/bt4187Isup2.hkl

Supporting information file. DOI: 10.1107/S2414314625009873/bt4187Isup3.cml

CCDC reference: 2500863

Additional supporting information:  crystallographic information; 3D view; checkCIF report

## Figures and Tables

**Table 1:** Hydrogen-bond geometry (Å, °)

*D*—H⋯*A*	*D*—H	H⋯*A*	*D*⋯*A*	*D*—H⋯*A*
C7—H7⋯N5^i^	0.95	2.44	3.2772 (19)	148

Symmetry code: (i) 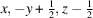.

**Table 2:** Experimental details

Crystal data	
Chemical formula	C_14_H_9_FN_2_O
*M*_r_	240.23
Crystal system, space group	Monoclinic, *P*2_1_/*c*
Temperature (K)	193
*a*, *b*, *c* (Å)	11.0938 (9), 8.6289 (3), 13.1513 (12)
β (°)	116.342 (4)
*V* (Å^3^)	1128.21 (15)
*Z*	4
Radiation type	Cu *K*α
μ (mm^−1^)	0.86
Crystal size (mm)	0.25 × 0.25 × 0.20

Data collection	
Diffractometer	Enraf–Nonius CAD-4
Absorption correction	–
No. of measured, independent and observed [*I* > 2σ(*I*)] reflections	2139, 2139, 1997
*R*_int_	0
(sin θ/λ)_max_ (Å^−1^)	0.610

Refinement	
*R*[*F*^2^ > 2σ(*F*^2^)], *wR*(*F*^2^), *S*	0.042, 0.124, 1.07
No. of reflections	2139
No. of parameters	164
H-atom treatment	H-atom parameters constrained
Δρ_max_, Δρ_min_ (e Å^−3^)	0.28, −0.28

Computer programs: *Enraf–Nonius Software* (Enraf–Nonius, 1989 ▸), *CORINC* (Dräger & Gattow 1971 ▸), *SHELXT2014* (Sheldrick, 2015*a* ▸), *SHELXL2019/3* (Sheldrick, 2015*b* ▸) and *PLATON* (Spek, 2009 ▸).

## full crystallographic data

### 4-[5-(4-Fluorophenyl)-1,2-oxazol-4-yl]pyridine Crystal data

**Table:**

C_14_H_9_FN_2_O	*F*(000) = 496
*M**_r_* = 240.23	*D*_x_ = 1.414 Mg m^−^^3^
Monoclinic, *P*2_1_/*c*	Cu *K*α radiation, λ = 1.54178 Å
*a* = 11.0938 (9) Å	Cell parameters from 25 reflections
*b* = 8.6289 (3) Å	θ = 65–69°
*c* = 13.1513 (12) Å	µ = 0.86 mm^−^^1^
β = 116.342 (4)°	*T* = 193 K
*V* = 1128.21 (15) Å^3^	Block, colourless
*Z* = 4	0.25 × 0.25 × 0.20 mm

### 4-[5-(4-Fluorophenyl)-1,2-oxazol-4-yl]pyridine Data collection

**Table:**

Enraf–Nonius CAD-4 diffractometer	*R*_int_ = 0
Radiation source: rotating anode	θ_max_ = 70.1°, θ_min_ = 4.5°
Graphite monochromator	*h* = −13→12
ω/2θ scans	*k* = 0→10
2139 measured reflections	*l* = 0→16
2139 independent reflections	3 standard reflections every 60 min
1997 reflections with *I* > 2σ(*I*)	intensity decay: 1%

### 4-[5-(4-Fluorophenyl)-1,2-oxazol-4-yl]pyridine Refinement

**Table:**

Refinement on *F*^2^	Hydrogen site location: inferred from neighbouring sites
Least-squares matrix: full	H-atom parameters constrained
*R*[*F*^2^ > 2σ(*F*^2^)] = 0.042	*w* = 1/[σ^2^(*F*_o_^2^) + (0.0738*P*)^2^ + 0.3458*P*] where *P* = (*F*_o_^2^ + 2*F*_c_^2^)/3
*wR*(*F*^2^) = 0.124	(Δ/σ)_max_ < 0.001
*S* = 1.07	Δρ_max_ = 0.28 e Å^−^^3^
2139 reflections	Δρ_min_ = −0.28 e Å^−^^3^
164 parameters	Extinction correction: SHELXL-2019/3 (Sheldrick, 2015b), Fc^*^=kFc[1+0.001xFc^2^λ^3^/sin(2θ)]^-1/4^
0 restraints	Extinction coefficient: 0.0086 (11)
Primary atom site location: structure-invariant direct methods	

### 4-[5-(4-Fluorophenyl)-1,2-oxazol-4-yl]pyridine Special details

**Table:**

Geometry. All esds (except the esd in the dihedral angle between two l.s. planes) are estimated using the full covariance matrix. The cell esds are taken into account individually in the estimation of esds in distances, angles and torsion angles; correlations between esds in cell parameters are only used when they are defined by crystal symmetry. An approximate (isotropic) treatment of cell esds is used for estimating esds involving l.s. planes.
Refinement. Hydrogen atoms were placed at calculated positions and were refined in the riding-model approximation with C–H = 0.95 Å, and with *U*_iso_(H) = 1.2 *U*_eq_(C).

### 4-[5-(4-Fluorophenyl)-1,2-oxazol-4-yl]pyridine Fractional atomic coordinates and isotropic or equivalent isotropic displacement parameters (Å^2^)

**Table:**

	*x*	*y*	*z*	*U*_iso_*/*U*_eq_	
F1	0.13611 (9)	0.46819 (11)	0.00038 (8)	0.0473 (3)	
O1	0.55369 (11)	0.27510 (14)	0.48748 (9)	0.0424 (3)	
C2	0.58461 (15)	0.33567 (17)	0.40706 (12)	0.0335 (3)	
C3	0.72080 (14)	0.33966 (16)	0.44657 (12)	0.0310 (3)	
C4	0.76924 (16)	0.27421 (18)	0.55716 (13)	0.0372 (4)	
H4	0.862023	0.259844	0.606378	0.045*	
N5	0.67381 (14)	0.23630 (18)	0.58372 (11)	0.0452 (4)	
C6	0.46742 (14)	0.37489 (17)	0.29991 (12)	0.0332 (3)	
C7	0.47353 (14)	0.35792 (18)	0.19678 (13)	0.0372 (4)	
H7	0.554463	0.323746	0.195893	0.045*	
C8	0.36212 (15)	0.39071 (19)	0.09582 (13)	0.0396 (4)	
H8	0.365560	0.380371	0.025244	0.047*	
C9	0.24600 (14)	0.43875 (17)	0.10017 (14)	0.0373 (4)	
C10	0.23548 (15)	0.45675 (18)	0.19966 (15)	0.0401 (4)	
H10	0.153807	0.490165	0.199504	0.048*	
C11	0.34809 (15)	0.42451 (18)	0.30042 (13)	0.0376 (4)	
H11	0.343831	0.436385	0.370570	0.045*	
C12	0.80557 (14)	0.39405 (16)	0.39348 (11)	0.0294 (3)	
C13	0.92774 (14)	0.32139 (17)	0.41942 (13)	0.0344 (3)	
H13	0.956804	0.237041	0.471192	0.041*	
C14	1.00631 (15)	0.37309 (19)	0.36917 (14)	0.0400 (4)	
H14	1.089593	0.322108	0.388691	0.048*	
N15	0.97329 (13)	0.48966 (17)	0.29514 (11)	0.0420 (4)	
C16	0.85625 (17)	0.55986 (19)	0.27201 (13)	0.0407 (4)	
H16	0.829864	0.643649	0.219731	0.049*	
C17	0.77120 (15)	0.51901 (17)	0.31884 (12)	0.0339 (3)	
H17	0.690549	0.575274	0.300406	0.041*	

### 4-[5-(4-Fluorophenyl)-1,2-oxazol-4-yl]pyridine Atomic displacement parameters (Å^2^)

**Table:**

	*U*^11^	*U*^22^	*U*^33^	*U*^12^	*U*^13^	*U*^23^
F1	0.0339 (5)	0.0487 (6)	0.0518 (6)	−0.0011 (4)	0.0122 (4)	0.0010 (4)
O1	0.0423 (6)	0.0510 (7)	0.0450 (6)	−0.0004 (5)	0.0296 (5)	0.0066 (5)
C2	0.0396 (8)	0.0324 (7)	0.0380 (8)	0.0001 (6)	0.0258 (7)	−0.0007 (6)
C3	0.0367 (7)	0.0277 (7)	0.0339 (7)	0.0019 (5)	0.0205 (6)	0.0004 (5)
C4	0.0433 (8)	0.0372 (8)	0.0369 (8)	0.0021 (6)	0.0230 (7)	0.0033 (6)
N5	0.0493 (8)	0.0521 (8)	0.0418 (8)	0.0038 (6)	0.0271 (7)	0.0093 (6)
C6	0.0327 (7)	0.0324 (7)	0.0400 (8)	−0.0020 (5)	0.0210 (6)	−0.0021 (6)
C7	0.0304 (7)	0.0441 (8)	0.0426 (8)	−0.0011 (6)	0.0212 (7)	−0.0054 (7)
C8	0.0371 (8)	0.0459 (9)	0.0402 (8)	−0.0047 (7)	0.0212 (7)	−0.0049 (7)
C9	0.0296 (7)	0.0339 (8)	0.0451 (8)	−0.0030 (6)	0.0135 (6)	−0.0008 (6)
C10	0.0339 (8)	0.0362 (8)	0.0574 (10)	0.0032 (6)	0.0266 (7)	−0.0001 (7)
C11	0.0395 (8)	0.0375 (8)	0.0454 (9)	0.0010 (6)	0.0276 (7)	−0.0014 (6)
C12	0.0327 (7)	0.0294 (7)	0.0298 (7)	−0.0036 (5)	0.0171 (6)	−0.0045 (5)
C13	0.0360 (7)	0.0322 (7)	0.0386 (8)	0.0018 (6)	0.0197 (6)	0.0000 (6)
C14	0.0346 (8)	0.0439 (9)	0.0472 (9)	−0.0005 (6)	0.0233 (7)	−0.0063 (7)
N15	0.0429 (7)	0.0493 (8)	0.0433 (7)	−0.0090 (6)	0.0278 (6)	−0.0052 (6)
C16	0.0461 (9)	0.0422 (8)	0.0376 (8)	−0.0048 (7)	0.0221 (7)	0.0038 (6)
C17	0.0341 (7)	0.0341 (7)	0.0354 (7)	0.0015 (6)	0.0173 (6)	0.0015 (6)

### 4-[5-(4-Fluorophenyl)-1,2-oxazol-4-yl]pyridine Geometric parameters (Å, º)

**Table:**

F1—C9	1.3614 (17)	C9—C10	1.373 (2)
O1—C2	1.3533 (17)	C10—C11	1.387 (2)
O1—N5	1.4118 (17)	C10—H10	0.9500
C2—C3	1.363 (2)	C11—H11	0.9500
C2—C6	1.471 (2)	C12—C13	1.391 (2)
C3—C4	1.425 (2)	C12—C17	1.393 (2)
C3—C12	1.4748 (18)	C13—C14	1.381 (2)
C4—N5	1.295 (2)	C13—H13	0.9500
C4—H4	0.9500	C14—N15	1.333 (2)
C6—C11	1.394 (2)	C14—H14	0.9500
C6—C7	1.395 (2)	N15—C16	1.340 (2)
C7—C8	1.384 (2)	C16—C17	1.382 (2)
C7—H7	0.9500	C16—H16	0.9500
C8—C9	1.378 (2)	C17—H17	0.9500
C8—H8	0.9500		
			
C2—O1—N5	109.06 (11)	C9—C10—C11	117.79 (14)
O1—C2—C3	109.53 (13)	C9—C10—H10	121.1
O1—C2—C6	114.50 (12)	C11—C10—H10	121.1
C3—C2—C6	135.92 (13)	C10—C11—C6	120.76 (14)
C2—C3—C4	103.39 (12)	C10—C11—H11	119.6
C2—C3—C12	131.26 (13)	C6—C11—H11	119.6
C4—C3—C12	125.35 (13)	C13—C12—C17	117.00 (13)
N5—C4—C3	113.09 (14)	C13—C12—C3	119.80 (13)
N5—C4—H4	123.5	C17—C12—C3	123.19 (13)
C3—C4—H4	123.5	C14—C13—C12	119.35 (14)
C4—N5—O1	104.93 (12)	C14—C13—H13	120.3
C11—C6—C7	119.52 (14)	C12—C13—H13	120.3
C11—C6—C2	120.23 (13)	N15—C14—C13	124.47 (14)
C7—C6—C2	120.22 (13)	N15—C14—H14	117.8
C8—C7—C6	120.21 (14)	C13—C14—H14	117.8
C8—C7—H7	119.9	C14—N15—C16	115.57 (13)
C6—C7—H7	119.9	N15—C16—C17	124.62 (15)
C9—C8—C7	118.36 (14)	N15—C16—H16	117.7
C9—C8—H8	120.8	C17—C16—H16	117.7
C7—C8—H8	120.8	C16—C17—C12	118.96 (14)
F1—C9—C10	118.64 (13)	C16—C17—H17	120.5
F1—C9—C8	118.01 (14)	C12—C17—H17	120.5
C10—C9—C8	123.35 (15)		
			
N5—O1—C2—C3	−0.45 (17)	C7—C8—C9—C10	0.5 (2)
N5—O1—C2—C6	177.46 (12)	F1—C9—C10—C11	179.28 (13)
O1—C2—C3—C4	0.79 (16)	C8—C9—C10—C11	0.0 (2)
C6—C2—C3—C4	−176.47 (16)	C9—C10—C11—C6	−0.3 (2)
O1—C2—C3—C12	−179.98 (14)	C7—C6—C11—C10	0.2 (2)
C6—C2—C3—C12	2.8 (3)	C2—C6—C11—C10	−177.78 (14)
C2—C3—C4—N5	−0.93 (18)	C2—C3—C12—C13	−147.29 (16)
C12—C3—C4—N5	179.79 (14)	C4—C3—C12—C13	31.8 (2)
C3—C4—N5—O1	0.66 (18)	C2—C3—C12—C17	34.1 (2)
C2—O1—N5—C4	−0.13 (17)	C4—C3—C12—C17	−146.80 (15)
O1—C2—C6—C11	32.3 (2)	C17—C12—C13—C14	−1.4 (2)
C3—C2—C6—C11	−150.50 (17)	C3—C12—C13—C14	179.94 (13)
O1—C2—C6—C7	−145.61 (14)	C12—C13—C14—N15	−0.5 (2)
C3—C2—C6—C7	31.6 (3)	C13—C14—N15—C16	1.3 (2)
C11—C6—C7—C8	0.3 (2)	C14—N15—C16—C17	−0.2 (2)
C2—C6—C7—C8	178.20 (14)	N15—C16—C17—C12	−1.7 (2)
C6—C7—C8—C9	−0.6 (2)	C13—C12—C17—C16	2.4 (2)
C7—C8—C9—F1	−178.88 (13)	C3—C12—C17—C16	−179.02 (13)

### 4-[5-(4-Fluorophenyl)-1,2-oxazol-4-yl]pyridine Hydrogen-bond geometry (Å, º)

**Table:**

*D*—H···*A*	*D*—H	H···*A*	*D*···*A*	*D*—H···*A*
C7—H7···N5^i^	0.95	2.44	3.2772 (19)	148

Symmetry code: (i) *x*, −*y*+1/2, *z*−1/2.

## References

[bb1] Abu Thaher, B., Koch, P., Schattel, V. & Laufer, S. (2009). *J. Med. Chem.* **52**, 2613–2617. 10.1021/jm801467h 19301816

[bb2] Dräger, M. & Gattow, G. (1971). *Acta Chem. Scand.* **25**, 761–762.

[bb3] Enraf–Nonius (1989). *CAD-4 Software Version 5*. Enra-f-Nonius, Delft, The Netherlands.

[bb4] Laufer, S. A., Margutti, S. & Fritz, M. D. (2006). *ChemMedChem* **1**, 197–207. 10.1002/cmdc.200500025 16892352

[bb5] Sheldrick, G. M. (2015*a*). *Acta Cryst.* A**71**, 3–8.

[bb6] Sheldrick, G. M. (2015*b*). *Acta Cryst.* C**71**, 3–8.

[bb7] Spek, A. L. (2009). *Acta Cryst.* D**65**, 148–155. 10.1107/S090744490804362X PMC2631630 19171970

